# miR-23a impairs bone differentiation in osteosarcoma via down-regulation of *GJA1*

**DOI:** 10.3389/fgene.2015.00233

**Published:** 2015-07-02

**Authors:** Yevgeniy Gindin, Yuan Jiang, Princy Francis, Robert L. Walker, Ogan D. Abaan, Yuelin J. Zhu, Paul S. Meltzer

**Affiliations:** ^1^Genetics Branch, Center for Cancer Research, National Institutes of HealthBethesda, MD, USA; ^2^Graduate Program in Bioinformatics, Boston UniversityBoston, MA, USA

**Keywords:** miR-23a, GJA1, bone, differentiation, osteosarcoma

## Abstract

Osteosarcoma is the most common type of bone cancer in children and adolescents. Impaired differentiation of osteoblast cells is a distinguishing feature of this aggressive disease. As improvements in survival outcomes have largely plateaued, better understanding of the bone differentiation program may provide new treatment approaches. The miRNA cluster miR-23a~27a~24-2, particularly miR-23a, has been shown to interact with genes important for bone development. However, global changes in gene expression associated with functional gain of this cluster have not been fully explored. To better understand the relationship between miR-23a expression and bone cell differentiation, we carried out a large-scale gene expression analysis in HOS cells. Experimental results demonstrate that over-expression of miR-23a delays differentiation in this system. Downstream bioinformatic analysis identified miR-23a target gene connexin-43 (*Cx43*/*GJA1*), a mediator of intercellular signaling critical to osteoblast development, as acutely affected by miR-23a levels. Connexin-43 is up-regulated in the course of HOS cell differentiation and is down-regulated in cells transfected with miR-23a. Analysis of gene expression data, housed at Gene Expression Omnibus, reveals that Cx43 is consistently up-regulated during osteoblast differentiation. Suppression of Cx43 mRNA by miR-23a was confirmed *in vitro* using a luciferase reporter assay. This work demonstrates novel interactions between microRNA expression, intercellular signaling and bone differentiation in osteosarcoma.

## 1. Introduction

Osteosarcoma is the most common primary bone malignancy and occurs most frequently in adolescents (Mirabello et al., 2009). Osteosarcoma tumors most often arise in the long bones of the skeleton, with more than half presenting around the knee (Broadhead et al., 2011), and is less common in axial skeleton (Martin et al., 2012). At diagnosis, 20% of osteosarcoma patients present with lung metastases with an additional 40% developing metastases at later stage (Martin et al., 2012). Survival rates for localized osteosarcoma are at 60–70% (Longhi et al., 2006; Mirabello et al., 2009), while the 5-year survival for osteosarcoma patients with metastases is 20% (PosthumaDeBoer et al., 2011). Despite intense research efforts, the survival rates for osteosarcoma have remained essentially unchanged for over two decades (Longhi et al., 2006; Mirabello et al., 2009). Contributing to the challenge of understanding and ultimately developing effective treatments for osteosarcoma is its complex karyotype and high level of chromosomal instability (Helman and Meltzer, 2003). With the increasing understanding of osteosarcoma biology, perturbation of cell differentiation is often regarded as aspect of this disease (Thomas and Kansara, 2006; Tang et al., 2008). Therefore, it is possible that the path toward developing new treatment approaches for osteosarcoma lies through an improved understanding of the dysregulation of the bone differentiation program in this devastating disease.

Discovery of small (about 22nt in length) non-coding RNA species, termed microRNAs (miRNAs), has, in many ways, revolutionized the understanding of gene expression regulation. It is now recognized, for instance, that miRNAs contribute to many biological processes (Ambros, 2004) and that their expression patterns can be used to classify cancers (Lu et al., 2005; Bloomston et al., 2007), suggesting that miRNAs play the roles similar to tumor suppressors and oncogenes (Dalmay and Edwards, 2006; Esquela-Kerscher and Slack, 2006). MicroRNAs play an integral role in controlling cell differentiation by suppressing genes that maintain plasticity (Yi et al., 2008), or by suppressing genes that inhibit cell-lineage commitment (Li et al., 2008) or through a combination of the two (Forrest et al., 2010).

miRNAs play a paramount role in bone differentiation (Sugatani and Hruska, 2007; Kobayashi et al., 2008; Wang et al., 2008; Inose et al., 2009; Hassan et al., 2012). Recent studies identified miRNA biomarkers relevant to therapy response and identification of therapeutic targets (Lulla et al., 2011; Maire et al., 2011; Jones et al., 2012; Cai et al., 2013). Much attention has been devoted to the role of miR-23a in bone differentiation, primarily via its targeting (both direct and indirect) of transcription factors essential to osteoblastogenesis such as TRPS1, RUNX2, and SATB2 (Hassan et al., 2010; Zhang et al., 2011, 2012). In the current work, we study the effects of miR-23a expression in HOS cells, which are distinguished from other human osteosarcoma cells by their ability to undergo a bone cell lineage differentiation program (Siggelkow et al., 1998; Hassan et al., 2010).

## 2. Materials and methods

### 2.1. Cell culture and bone differentiation

All cell lines were obtained from ATCC. The cells were grown in DMEM media with 10% fetal bovine serum and supplemented with 1% penicillin and streptomycin. HOS cells were grown to got 100% confluence, followed by differentiation at 7–9 days induced by bone inducing agents, that include L-ascorbic acid 50 ug/ml and beta-glycerophosphate 5 mM (Hassan et al., 2006). Cells were harvested at indicated times for mRNA and protein extraction or fixed with 10% neutral-buffered formalin (NBF) for detection of calcium deposits by Alizarin Red staining.

### 2.2. RNA analyses

Total RNA was isolated using Trizol reagent (Invitrogen), treated with DNase I (Ambion) and reverse transcribed using “iScript Reverse Transcription Supermix for RT-qPCR” (BIO-RAD). *GJA1* and *COL1A1* gene expression qRT-PCR were performed using the TaqMan Gene Expression Assays (ABI/ Life Technologies). mRNA levels were normalized to housekeeping gene ACTB. miRNA-23a was quantified in triplicate using the TaqMan MicroRNA Assay (ABI/ life technologies) and normalized to U6. mRNA levels were assayed for relative expression using procedure described in Livak and Schmittgen (2001).

### 2.3. Illumina expression arrays

HOS cells were transfected with human hsa-miR-23a, or negative control mimic (Thermo Scientific) for 72 h. 150 ng of each RNA was amplified and labeled using the “Illumina TotalPrep RNA Amplification kit" (LifeTechnologies). The biotin-labeled cRNAs were quantitated spectrophotometrically and 750 ng was hybridized to Illumina HumanRef-8v3 Expression BeadChip microarrays (Illumina, San Diego, CA). BeadChips were scanned in an Illumina Scanner. The data has been deposited in the GEO database under accession number. One SuperSeries record GSE68014; Two regular Series records: GSE68012 and GSE68012.

### 2.4. Protein immunoblot analyses

Whole cell lysates from transfected HOS cells were prepared using RIPA buffer. Proteins were analyzed by SDS PAGE, transferred to nitrocellulose membranes and probed with GJA1 antibody (ab11370 Abcam). Western Blots were quantified by densitometry.

### 2.5. Luciferase reporter assay

HOS cells were co-transfected in 24 well-plates using Lipofectamine 2000 (Invitrogen) with 20 nM miR-23a mimic or control miRNA mimic and 100 ng of psiCHECK2- 3UTR (Promega) vector containing the GJA1-3UTR cloned into the multiple cloning site of Renilla luciferase. After 48 h of transfection luciferase activity was measured using the Dual Luciferase Assay System (Promega). The experiment was performed in triplicate. Results were normalized to those obtained in cells transfected with an empty vector. Data were normalized to Firefly luciferase and results from 3 independent experiments were compared. GJA1 sequences were cloned into psiCHECK-2 by annealing complementary oligomers matching each GJA1 sequence with overhanging ends complementary to the XhoI and NotI sites of psiCHECK-2.

### 2.6. siGJA1 transfection assay

HOS cells were differentiated as described above. One day after induction of differentiation, cells were transfected using Lipofectamine RNAiMAX Reagent (Invitrogen) with ON-TARGETplus-siGJA1-pool, siGJA1-05, siGJA1-06 (Thermo Scientific L-011042-00-0005) at a final concentration of 100 pmol. After 72 h transfection, on differentiation day 4, the cells were harvested for mRNA, protein assays, and ALP activity assay or fixed with 10% NBF for detection of calcium deposits by Alizarin Red Staining.

### 2.7. ALP assay in siGJA1-transfected HOS cells

Alkaline phosphatase activity was determined in HOS cell lysates using the colorimetric Alkaline Phosphatase Assay Kit (Abcam, Cat No: ab83369). The kit uses *p*-nitrophenyl phosphate as a phosphatase substrate, which turns yellow when dephosphorylated by alkaline phosphatase. The absorbance at 405 nm was measured using a multi well plate reader (550 Microplate Reader; Bio-Rad Laboratories). Each assay condition was carried out in triplicate. Cell lysates were analyzed for protein content using the Bio-Rad DC Protein Assay (Bio-Rad Laboratories), and alkaline phosphatase activity was normalized for total protein concentration.

### 2.8. Alizarin red staining in siGJA1 transfected HOS cells

HOS cells were fixed with 10%NBF^*^(10%Formalin solution, neutral buffered, SIGMA HT501128-4L) on differentiation day 4 with GJA1 silencing 72 h, followed by “Alizarin Red S Staining” (SIGMA A5533-25G) using NovaUltra Special Stain Kits protocol. The red staining is indicative of calcium deposits.

### 2.9. Data analysis

All statistical analyses were carried out using the R statistical environment version 3.0. Microarray data were analyzed using limma package (Smyth, 2005). Data from GEO were obtained using the GEOquery package (Davis and Meltzer, 2007).

## 3. Results

### 3.1. Induction of differentiation in osteosarcoma cells

**Figure 1:**
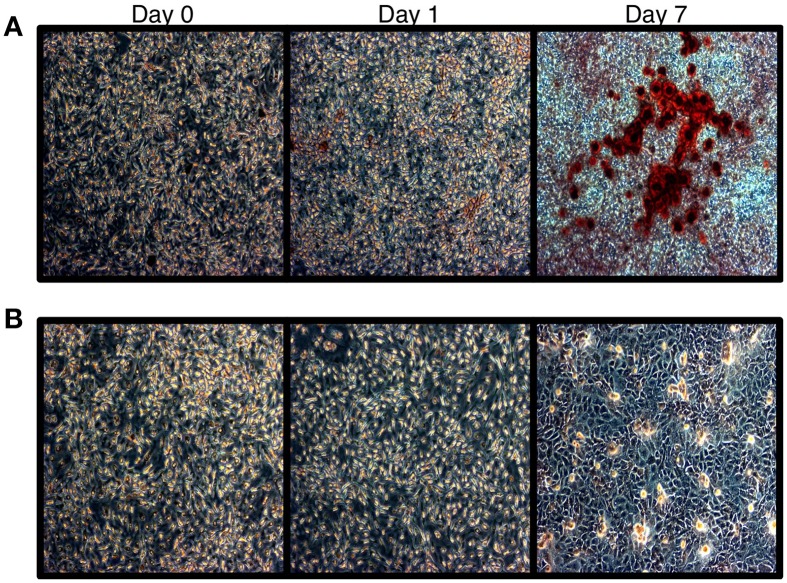
**Alizarin red staining of HOS cells. (A)** Alizarin red staining of HOS cells during the differentiation time course. Red staining is indicative of calcium deposits. **(B)** Untreated cells.

**Figure 2:**
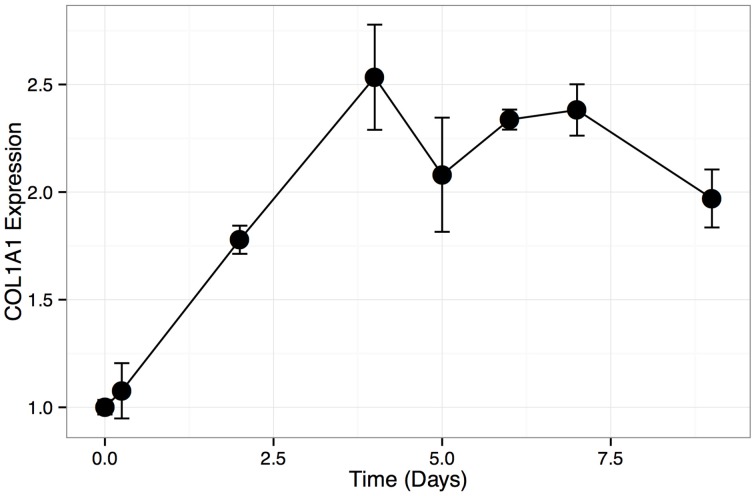
**COL1A1 expression in HOS differentiation time course**. Relative expression levels (assayed with qPCR) of COL1A1 during HOS differentiation time course. Expression levels are normalized to the initial time point. Error bars represent standard deviation.

To confirm that HOS cells are amendable to bone differentiation induction (Siggelkow et al., 1998), we treated these cells with L-ascorbic acid, which induces the formation of collagenous extracellular matrix and brings an osteoblast-specific gene expression program in osteogenic lineage cells (Franceschi et al., 1994). We then monitored HOS cell culture for the presence of calcium deposits, which serve as a marker of bone mineralization, via Alizarin Red staining. Our results indicate that HOS cells undergo osteoblast-like differentiation upon stimulation with L-ascorbic acid. HOS cells exhibit intense Alizarin Red staining on day 7 post differentiation induction (Figure 1). We confirmed this result by monitoring the expression of collagen Ia1 (*COL1A1*)—a gene marker of bone differentiation (Figure 2). We have observed a two-fold increase in *COL1A1* mRNA levels between the initial and terminal differentiation time-points.

### 3.2. miR-23a targets genes involved in bone differentiation

To study the effect of miR-23a on the gene expression program in osteosarcoma, we set out to identify genes that are transcriptionally repressed by miR-23a in HOS cells. To that end, we first transfected HOS cells with a miR-23a mimic and compared their gene expression profile with mock-transfected cells using Illumina microarrays. Our analysis shows that 1530 genes (see Supplemental Table 1) are down-regulated in miR-23a transfected cells vs. mock-transfected cells. These genes are significantly enriched for predicted (Lewis et al., 2005) miR-23a targets (262 overlapping genes (see Supplemental Table 2); *P_overlap_* = 4.96 × 10^−46^).

Having established the on-target effects of miR-23a over-expression in HOS cells, we next asked if the genes affected by miR-23a overlap significantly with genes that change in expression during HOS differentiation. Given miR-23a's role as a dampener of bone differentiation gene expression program (Hassan et al., 2010), we focused on genes that are up-regulated during the HOS differentiation time course. To that end, we compared mRNA expression levels in HOS cells prior to differentiation induction with that of HOS cells that display phenotypic properties of bone cells post differentiation induction using microarrays in triplicate. Our analysis shows that 3065 genes (see Supplemental Table 3) increase in expression during HOS cell differentiation. Of those, 466 genes (see Supplemental Table 4) are down-regulated upon miR-23a transfection (*P_overlap_* = 1.21 × 10^−10^). To identify genes of interest that are under miR-23a control and are relevant to HOS cell differentiation, we identified 77 genes (see Supplemental Table 5) that meet the following criteria: (i) are computationally predicted miR-23a targets; (ii) are down- regulated on miR-23a over-expression and; (iii) are up-regulated during HOS cell differentiation time course.

Transcription factor binding site enrichment analysis (Loots et al., 2002) reveals that more than one-half of the 77 genes contain an SP1 transcription factor motif within 2 kb of their transcription start site [42 genes (see Supplemental Table 6); *P_enrichment_* = 1 × 10^−23^]. These results suggest that over-expression of miR-23a interferes with the bone differentiation program by counteracting the action of osteoblast lineage inducing transcription factors binding to the SP1 motif. The SP1 transcription factor family include osterix (SP7), a bone lineage specific factor required for osteoblast differentiation and bone formation (Nakashima et al., 2002). These results suggest that over-expression of miR-23a interferes with the bone differentiation program by counteracting the action of osteoblast lineage inducing transcription factor SP1. In order to narrow down the list of likely miR-23a targets that are involved in bone differentiation we looked for gene signature enrichments, as curated in Molecular Signatures Database (Subramanian et al., 2005), among the 42 high-quality miR23a down-regulated genes with an upstream SP1 binding site. The top-enriched signature is that of genes up-regulated upon *EZH2* knock-down in prostate cancer cells (Nuytten et al., 2008) (Table 1; *P_overlap_* = 3.61 × 10^−9^). EZH2, the catalytic subunit of the PRC2 repressive complex, is commonly associated with silencing of pro-differentiation genes (Simon and Lange, 2008), a function analogous with that of miR-23a in bone differentiation. This observation is particularly interesting as prostate metastatic tumors are often osteoblastic (Logothetis and Lin, 2005) and phosphorylation of EZH2 by CDK1 is critical for osteogenic differentiation of human bone-marrow-derived mesenchymal cells (Wei et al., 2011).

**Table 1:** **miR-23a Target Genes Relevant to HOS Differentiation**.

**Gene symbol**	**Gene name**
CAB39	Calcium binding protein 39
CLDN12	Claudin 12
DCBLD2	Discoidin, CUB and LCCL domain containing 2
FAM46A	Family with sequence similarity 46, member A
GJA1	Gap junction protein, alpha 1, 43kDa (connexin 43)
IRF1	Interferon regulatory factor 1
MARCKS	Myristoylated alanine-rich protein kinase C substrate
RAB8B	RAB8B, member RAS oncogene family
TNFAIP3	Tumor necrosis factor, alpha-induced protein 3
UBL3	Ubiquitin-like 3

### 3.3. GJA1 is a major target of miR-23a

In order to identify a specific miR-23a target related to bone differentiation, we examined the gene list in Table 1 for genes involved in sensing extracellular environment and intercellular communication, which are essential in bone formation that are also present in the aforementioned gene signatures. Expert-based examination of the gene list, through the use of manual curation and gene ontology functionality related to bone differentiation, led a gene whose product is connexin(Cx)-43 (also known as *GJA1*). GJA1 is a member of the gap junction family and is the most abundant gap junction expressed in bone (Loiselle et al., 2013), where it facilitates response to extracellular mechanical (Jiang et al., 2007), pharmacologic and hormonal stimuli (Plotkin and Bellido, 2013) and is required for signal transduction among bone lineage cells (Civitelli, 2008). Crucially, GJA1 is essential for osteoblast differentiation in humans and animals *in vivo* (Stains and Civitelli, 2005a).

**Figure 3:**
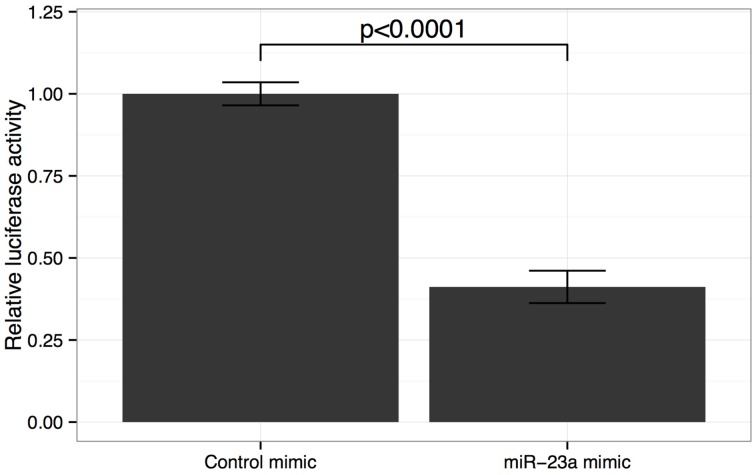
**Effect of miR-23a mimic on GJA1 3′ UTR luciferase activity**. HOS cells were co-transfected with either miR-23a mimic or control miRNA mimic and psiCHECK2- 3UTR vector containing the GJA1-3UTR of Renilla luciferase. Data were normalized to Firefly luciferase activity and cells transfected with empty psiCHECK-2 control vectors. Error bars represent standard deviation of technical repeat experiments (*n* = 3). *P*-value calculated by Student's *t*-test.

We verified that the miR-23a binding site is well-conserved in 3′UTR of the GJA1 gene (Lewis et al., 2005; Friedman et al., 2009). We next sought to verify miR23a:*GJA1* interaction *in vitro*. To that end we carried out a reporter assay where the 3′UTR of *GJA1* was cloned into the 3′UTR of a luciferase gene. We find that miR-23a significantly reduces luciferase *GJA1* reporter activity (Figure 3). These results confirm that *GJA1* is a bona fide miR-23a target.

We then set out to elucidate the expression pattern of *GJA1* during osteoblast cell differentiation. To that end, we identified, in Gene Expression Omnibus (GEO) (Barrett et al., 2013), a dataset used in two recently published studies (Nabavi et al., 2012; Pustylnik et al., 2013) that assayed gene expression in mouse MC3T3-E1 osteoblast cells following differentiation induction by L-ascorbic acid. Neither study explicitly addressed gap junction expression in osteoblast differentiation. Our analysis of the data deposited in GEO shows that *GJA1* expression increases over 500-fold (6-probe average; *P* < 1.0 × 10^−6^) in mouse osteoblast cells following differentiation.

**Figure 4:**
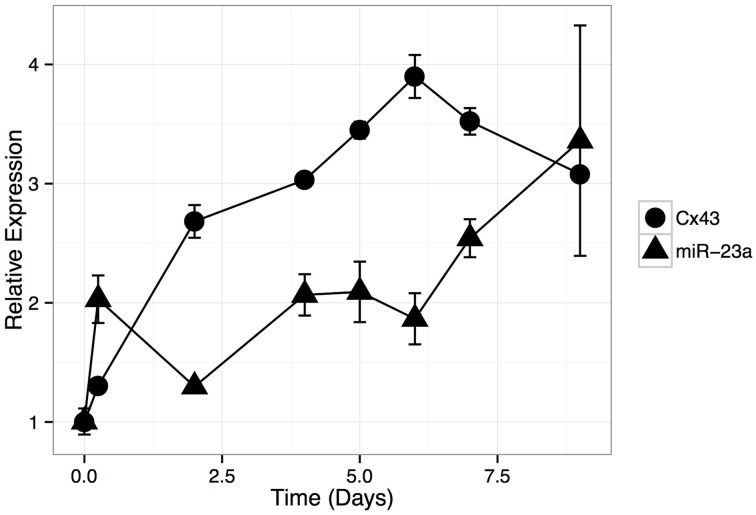
**Relative expression of GJA1 and miR-23a during HOS cell differentiation time course**. HOS cells were induced to differentiate and mRNA aliquots were isolated at selected time points (x-axis). Relative expression of GJA1 and miR-23a were normalized, separately, to their basal levels (Day 0). Error bars represent standard deviation due to technical repeats (*n* = 3).

Next, we asked whether *GJA1* expression levels increase during HOS cell differentiation and how this expression pattern may be related to miR-23a expression. To that end, we induced differentiation in HOS cells and analyzed mRNA expression with quantitative (q)PCR. Our results show (Figure 4) that *GJA1* levels increase as HOS cells begin to display phenotypical hallmarks of osteblast cells. *GJA1* levels reach their maximum on day 7 post differentiation induction, at which point HOS cells display the phenotypic properties of bone differentiation (Figure 1) and *COL1A1* levels have reached their peak (Figure 2). Importantly, miR-23a levels are inversely related to *GJA1* expression: reaching minimum, when *GJA1* levels are at their peak and Alizarin red staining is at its maximum; then increasing gradually past day 4 when *GJA1* levels off and begins to decrease.

### 3.4. Knock-down of GJA1 delays HOS cell differentiation

**Figure 5:**
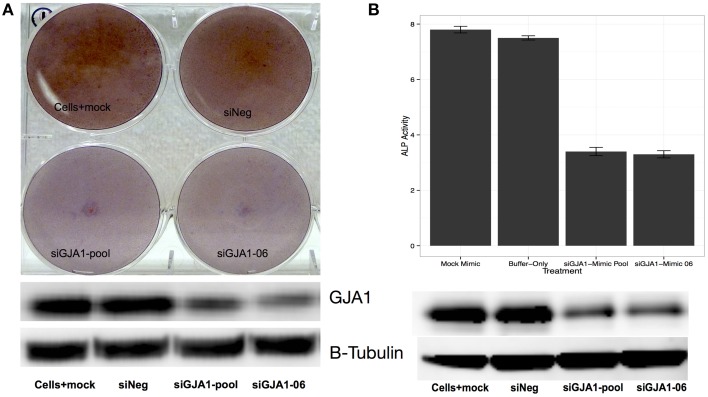
**Effect of** ***GJA1*** **knock-down on HOS cell differentiation**. HOS cells were induced to differentiate and were then transfected with either: (i) a scrambled siRNA negative control, or (ii) empty buffer negative control, or (iii) a pool of GJA1 siRNA constructs, or (iv) a single GJA1 siRNA construct number 6. 72 h after transfection cells were either **(A)** stained with Alizarin Red, or **(B)** subjected to measurement of alkaline phosphatase (ALP) activity. In both cases, an aliquot of cells was used to measure to GJA1 protein concentration via Western blot (bottom panels images). Error bars represent standard error from the mean from three replicates.

We next examined the relationship between *GJA1* expression and HOS cell differentiation (Figure 5). To that end, we once again induced HOS cell differentiation, but this time transfecting HOS cells with GJA1 siRNA constructs 24 h after differentiation induction. First, we examined the *GJA1* knock-down effect on differentiation qualitatively via Alizarin Red staining (Figure 5A). As shown in Figure 5A, cells that were transfected with GJA1 siRNA produced less extracellular calcium deposits (as evidence by stain intensity) compared to either to either of the negative controls. Lastly, we sought to quantitatively measure the effect of *GJA1* knock-down on HOS cell differentiation. To that end we measured alkaline phosphatase (ALP) activity in these cells after differentiation induction and transfection with either GJA1 siRNA or negative controls. As shown in Figure 5B, there is a more than a two-fold reduction in ALP activity in HOS cells where *GJA1* is knocked-down in siRNA. Importantly, GJA1 siRNA transfection led to decreased GJA1 protein levels in both of these experiments (Figure 5). Together, these results demonstrate that reduction of *GJA1* expression prevents HOS cells from displaying matrix calcification phenotype and ALP activity that is associated with osteoblast cells (Figure 5).

## 4. Discussion

A number of recent studies have identified miRNAs that are differentially expressed between normal bone and osteosarcoma (see recent summaries in Miao et al., 2013 and Zhou et al., 2013). Importantly, miR-23a has been shown to control bone differentiation (Hassan et al., 2010; Zhang et al., 2011, 2012). However, it is not clear what role, if any, miR-23a has within the realm of osteosarcoma. Studies that focused on the function of miR-23a in bone differentiation have been restricted to the examination of miR-23a and expression of transcription factors that are paramount to bone biology such as RUNX2 (Zhang et al., 2011, 2012) and/or SATB2 (Hassan et al., 2010), which were chosen *a priori*. We find that miR-23a acts to inhibit differentiation, at least in part, by blocking the expression of connexin 43 (GJA1) a key protein for of cell-cell communication and osteoblast differentiation.

In this study we examined the relationships between miR-23a and bone differentiation within the context of osteosarcoma. Previous studies demonstrated the relationship between miR-23a and transcription factors that are central to bone differentiation program (Hassan et al., 2010; Zhang et al., 2012). Separately, Inose et al. (2009) have shown that bone differentiation is negatively impacted in mice by miR-206-mediated silencing of *GJA1*. We could not detect miR-206 expression in HOS nor human osteoblast cells (data not shown). This points to redundant pathways that fine-tune *GJA1* expression during bone differentiation.

Loss of gap junctional communication delays osteoblast differentiation and reduces the ability of these cells to form mineralized extracellular matrix (Lecanda et al., 1998; Schiller et al., 2001). A clue as to how this effect arises came from an observation that loss of GJA1 function is accompanied by diminished extracellular-signal-regulated kinase (ERK) activity (Stains and Civitelli, 2005b). In the proposed mechanism, a GJA1 gap junction allows for passage of a 2nd messenger activating ERK/PI3K signaling cascades that would in turn recruit transactivator SP1 to promoter regions of genes associated with the osteoblastic gene expression program, such as osteocalcin and *COLIA1* (Stains et al., 2003; Stains and Civitelli, 2005b). The loss of GJA1 gap junctions diminishes ERK activity resulting in preferential recruitment of SP3 repressor to osteocalcin and *COLIA1* gene promoters (Stains et al., 2003; Stains and Civitelli, 2005b). Here, we show that miR-23a gene targets in HOS cells are enriched for SP1 binding site within 2kb of their transcription start site, which suggests that miR-23a may function by counteracting the effects of the SP1 family of transcription factors.

## Author contributions

YG and YJ wrote the manuscript. YJ, PF, and RW performed experiments. YG and YZ analyzed data. PM and PF conceived the study. PM supervised the work and edited the manuscript.

### Conflict of interest statement

The authors declare that the research was conducted in the absence of any commercial or financial relationships that could be construed as a potential conflict of interest.
